# Placing vacuum sponges in esophageal anastomotic leaks — how we do it

**DOI:** 10.1007/s00423-024-03272-5

**Published:** 2024-03-05

**Authors:** Florian Hentschel, Götz Mollenhauer, Björn Siemssen, Christoph Paasch, René Mantke, Stefan Lüth

**Affiliations:** 1 Brandenburg Medical School (Theodor Fontane), Brandenburg, Germany; 2 Klinik für MIC, Berlin, Germany; 3 Shouldice Hospital, Thornhill, ON Canada; 4 Zentrum für Innere Medizin II, Hochschulklinikum Brandenburg der MHB, Hochstr. 29, 14770 Brandenburg an der Havel, Germany

**Keywords:** Esophagus, Esophagogastric anastomosis, Anastomotic leak, Vaccum therapy, Eso-Sponge®

## Abstract

**Purpose:**

Endoluminal vacuum sponge therapy has dramatically improved the treatment of anastomotic leaks in esophageal surgery. However, the blind insertion of vacuum sponge kits like Eso-Sponge® via an overtube and a pusher can be technically difficult.

**Methods:**

We therefore insert our sponges under direct visual control by a nonstandard “piggyback” technique that was initially developed for the self-made sponge systems preceding these commercially available kits.

**Results:**

Using this technique, we inserted or changed 56 Eso-Sponges® in seven patients between 2018 and 2023. Apart from one secondary sponge dislocation, no intraprocedural complications were encountered. One patient died due to unrelated reasons. In all others, the defects healed and they were dismissed from the hospital. Long-term follow-up showed three strictures that were successfully treated by dilatation.

**Conclusion:**

We conclude that sponge placement via piggyback technique is a fast, safe, and successful alternative to the standard method of insertion.

## Introduction

A frequent problem in esophageal surgery is the weakness of the esophago-gastric or esophago-jejunal anastomosis [1, 2]. Unlike in other anastomoses in the GI tract, leakage rates of up to 25% are the rule rather than the exception here [3, 4]. Moreover, for a long time all methods of treating these leaks, like early re-operation, endoscopic clipping, or stent insertion, were rather unsuccessful and frustrating [5]. This situation changed dramatically with the introduction of endo-luminal vacuum therapy in the upper GI tract in 2007 [6–8].﻿  And even if there are no large head-to-head studies yet, the superiority of this principle has since been proven [9, 10]. 

In the first years after the introduction of vacuum therapy in the upper GI tract, endoscopists used self-made systems [6]. These usually consisted of a sponge intended for cutaneous wounds that were cut to size and sewn to a nasogastric tube [11, 12]. Since 2014, these devices were superseded by commercially available endoluminal vacuum sponge systems that were designed and approved for the use in the upper GI tract (Eso-Sponge®, B. Braun, Germany) [13]. In these systems, the tube and sponge come in one piece, but they are mostly of the same form factor as the old ones. The main difference is the method of insertion: While the old systems were introduced via the piggyback technique (see below), the new ones consist of the sponge system itself, an overtube, and a pusher. In brief, the overtube is put over the gastroscope and placed inside the cavity, the scope is then removed, and the sponge is advanced by a special pusher that has a longitudinal lumen for the vacuum line. Once a mark is reached that indicates that the sponge and the tip of the overtube align, the pusher will be fixated and the overtube slowly retracted. The sponge is thus released inside the cavity. The overtube and pusher are then removed [14].

This method was originally developed for the rectum, where the way to the anastomosis is straight and short, the lumen is wide, and the abscess cavity is often large [15]. In the upper GI tract, however, the esophageal lumen is considerably smaller, access through the pharynx is curved, and the upper esophageal sphincter can be tight. All this may render it difficult or even dangerous to insert the relatively stiff and thick overtube. Moreover, once the overtube has been placed, the sponge will be pushed blindly forward through the curve, bearing the risk of dislocation. We therefore adapted the original piggyback method to be used with the Eso-Sponge® System and leave out the overtube completely.

## How we do it

**Fig. 1:**
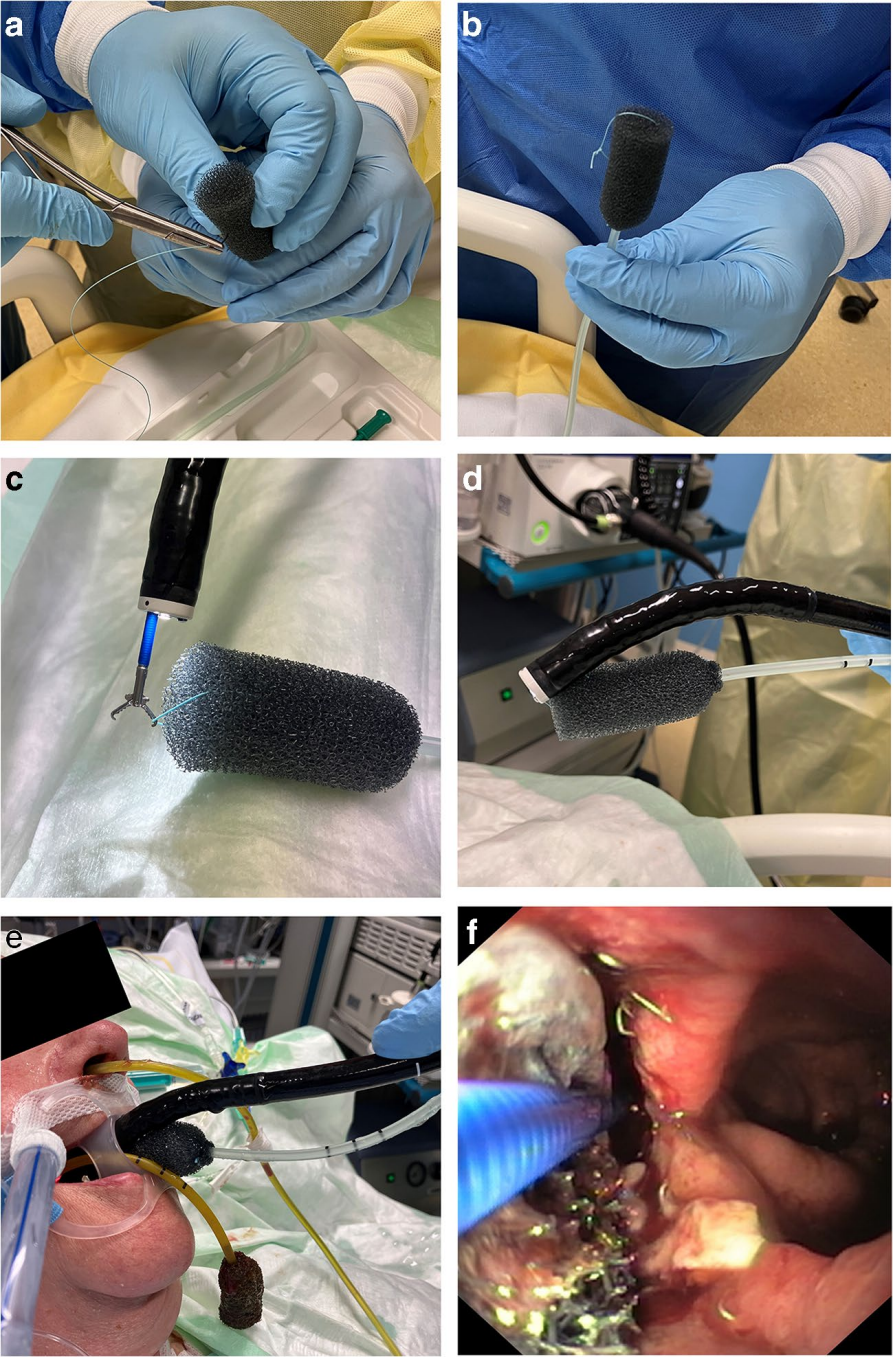
Piggyback technique for sponge insertion or changing in six steps. **A**, **B** When the air knot is applied to the vacuum tube, care should be taken that the suture really goes through the tube and not just through the sponge. **F** Gastric lumen in the right of the picture, anastomotic leak on the left. The sponge is brought halfway into the cavity with forceps still attached to the knot. For all other phases, see text

After unpacking the Eso-Sponge®, a needle with a 2–0 suture is placed through the tip of the vacuum tube (Fig. 1A), and a small air knot is tied (Fig. 1B). This knot is grabbed with forceps (Fig. 1C), and pulled just deep enough into the working canal that the tips of the scope and the sponge align (Fig. 1D). After thorough lubrication, the scope and sponge are then brought into the patient via a piggyback technique (Fig. 1E). Often, a certain resistance in the upper esophageal sphincter will be felt. But since patients are generally sedated, and many are mechanically ventilated, this resistance can be overcome. The scope with the sponge will then easily be advanced into or in front of the cavity. Now, the forceps are pushed forward while the scope is pulled back. In this way, the sponge can be placed exactly into its desired position under full visual control (Fig. 1F). Finally, the forceps are opened and freed from the suture by a short wiggling motion, and the scope and forceps are removed. (We apply a little back-and-forth rotation while removing the scope to ensure that there is sliding friction rather than static friction between the scope and the vacuum tube all of the time.)

After the initial placement or changing of the sponge, the vacuum tube is then brought from an esophago-oral to an esophago-nasal position in the usual way. The only difference in our clinic is that during changes, we usually attach the new vacuum tube to the old one and use this as a lead when pulling it through the pharynx and nose (Fig. 1E).

## Results

Between January 2016 and June 2023, our clinic treated seven patients with Eso-Sponges® using this technique. Three patients were female, four were male; mean age was 61 years. Six had developed anastomotic leaks after partial gastrectomy for carcinoma; one suffered from a perforation during a hiatal hernia repair. The median time from OP to the first sponge insertion was 6 days. In six patients, the leak was big enough that the sponge could initially be inserted into a necrotic cavity; in one patient, the leak was so small that the sponge was placed in the esophageal lumen. Sponges were changed by the same piggyback technique every 3 to 6 days. Thus, a total of seven initial sponge insertions, and 49 consecutive sponge changes were carried out. We observed two intraprocedural complications: One bleeding after sponge removal that stopped spontaneously, and one dislocated sponge that was found in the stomach during the next endoscopy. It was removed and a new sponge was placed into the cavity without any problem (Table 1). One patient died due to peritonitis and septicemia from a leaking jejunal catheter that was not related to the esophageal anastomosis. The six others could be weaned and were finally dismissed after the leaks has healed. Long-term follow-up found three strictures that were successfully treated by dilatation.

**Table 1:** Patients, vacuum sponge procedures, complications, and outcome

Pat. No	Sex	Age	Diagnosis, OP	Days between OP and first sponge insertion	Initial placement	Number of Sponge changes	Intraprocedural complications	Primary outcome
1	W	81	Adenocarcinoma of esophagogastric junction	2	Cavity	8	None	Died, unrelated
2	M	65	Adenocarcinoma of esophagogastric junction	8	Lumen	4	None	Remission
3	W	58	Proximal gastric carcinoma	6	Cavity	4	None	Remission, stricture
4	M	61	Barrett’s carcinoma	6	Cavity	15	None	Remission, stricture
5	W	74	Paraesopahgeal hernia; perforation of esophagus during hiatoplasty	1	Cavity	5	None	Remission
6	M	54	Adenocarcinoma of esophagogastric junction	14	Cavity	11	1st: Bleeding; 6th dislocation (stomach)	Remission
7	M	61	Adenocarcinoma of esophagogastric junction	15	Cavity	2	None	Remission, stricture

## Discussion

Vacuum sponge therapy has been proven a safe and successful treatment for anastomotic leaks in the upper and lower GI tract [15, 16]. In esophageal surgery, where anastomoses are prone to insufficiencies and leakage, it has been no less than a game changer [9, 10].

Since the therapy is relatively new, the development of the technique is still ongoing [6, 17, 18]. So even if there is a commercial “all-in-one” system available now, alternative methods for sponge insertion are still under development, and a “freestyle” approach that is adapted to existing anatomic peculiarities can still be beneficial [19]. We therefore wanted to share our own experience with a less traumatic sponge insertion method that is nonstandard but, in our opinion, easier and at least not inferior to the one proposed by the manufacturer of the device [14].

Using this piggyback technique, we inserted 56 sponges within 7 years (Table). In these, we observed two complications: One bleeding after removal of the old sponge that is most likely unrelated to the method of insertion and would have occurred anyway. The other was a dislocation of the sponge into the stomach. We cannot rule out that this was due to the insertion method, but as the initial photo documentation showed a correct position in the cavity, it could also be secondary due to nursing maneuvers or spontaneous movements of the patient.

In summary, our experience with Eso-Sponge® insertion via piggyback technique has been positive, and we encourage all colleagues who are uncomfortable with the standard method to consider it as an alternative.

## Data Availability

All relevant data are within the paper.
